# 2-[4-(2-Formyl­phen­oxy)­but­oxy]­benzaldehyde

**DOI:** 10.1107/S1600536811034210

**Published:** 2011-08-27

**Authors:** Aliakbar Dehno Khalaji, Salar Hafez Ghoran, Kazuma Gotoh, Hiroyuki Ishida

**Affiliations:** a Department of Chemistry, Faculty of Science, Golestan University, Gorgan, Iran; b Department of Chemistry, Faculty of Science, Okayama University, Okayama 700-8530, Japan

## Abstract

In the crystal structure of the title compound, C_18_H_18_O_4_, the full mol­ecule is generated by the application of an inversion centre. The mol­ecule is essentially planar, with an r.m.s. deviation of 0.017 (1) Å for all non-H atoms. The mol­ecules are linked through inter­molecular C—H⋯O inter­actions to form a mol­ecular sheet parallel to the (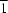
02) plane.

## Related literature

For the synthesis and related structures, see: Hu *et al.* (2005 ▶); Aravindan *et al.* (2003 ▶). For related literature on Schiff bases and their transition metal complexes, see: Ilhan *et al.* (2009 ▶, 2010 ▶); Yilmaz *et al.* (2009 ▶).
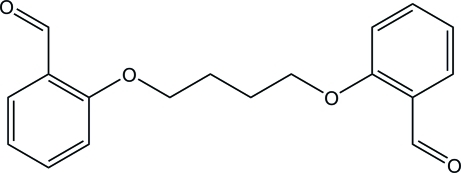


## Experimental

### 

#### Crystal data

C_18_H_18_O_4_
*M* *_r_* = 298.34
Monoclinic, 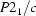

*a* = 8.0624 (7) Å
*b* = 14.5896 (7) Å
*c* = 6.8003 (4) Å
β = 108.549 (4)°
*V* = 758.35 (8) Å^3^
*Z* = 2
Mo *K*α radiation
μ = 0.09 mm^−1^
*T* = 190 K
0.30 × 0.24 × 0.15 mm

#### Data collection

Rigaku R-AXIS RAPID II diffractometer
Absorption correction: numerical (*NUMABS*; Higashi, 1999 ▶) *T*_min_ = 0.980, *T*_max_ = 0.986
12149 measured reflections
2210 independent reflections
1243 reflections with *I* > 2σ(*I*)
*R*_int_ = 0.041

#### Refinement

*R*[*F* ^2^ > 2σ(*F* ^2^)] = 0.044
*wR*(*F* ^2^) = 0.132
*S* = 1.13
2210 reflections
100 parameters
H-atom parameters constrained
Δρ_max_ = 0.29 e Å^−3^
Δρ_min_ = −0.24 e Å^−3^

### 

Data collection: *PROCESS-AUTO* (Rigaku/MSC, 2004 ▶); cell refinement: *PROCESS-AUTO*; data reduction: *CrystalStructure* (Rigaku/MSC, 2004 ▶); program(s) used to solve structure: *SHELXS97* (Sheldrick, 2008 ▶); program(s) used to refine structure: *SHELXL97* (Sheldrick, 2008 ▶); molecular graphics: *ORTEP-3* (Farrugia, 1997 ▶); software used to prepare material for publication: *CrystalStructure* and *PLATON* (Spek, 2009 ▶).

## Supplementary Material

Crystal structure: contains datablock(s) global, I. DOI: 10.1107/S1600536811034210/tk2783sup1.cif

Structure factors: contains datablock(s) I. DOI: 10.1107/S1600536811034210/tk2783Isup2.hkl

Supplementary material file. DOI: 10.1107/S1600536811034210/tk2783Isup3.cml

Additional supplementary materials:  crystallographic information; 3D view; checkCIF report

## Figures and Tables

**Table 1:** Hydrogen-bond geometry (Å, °)

*D*—H⋯*A*	*D*—H	H⋯*A*	*D*⋯*A*	*D*—H⋯*A*
C3—H3⋯O1^i^	0.95	2.53	3.397 (2)	152

Symmetry code: (i) 
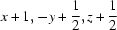
.

**Fig. 1.**
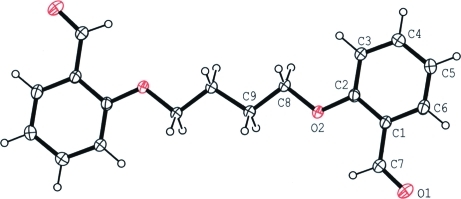
The molecular structure of the title compound, showing the atom-numbering scheme. Displacement ellipsoids of non-H atoms are drawn at the 50% probability level.

**Fig. 2.**
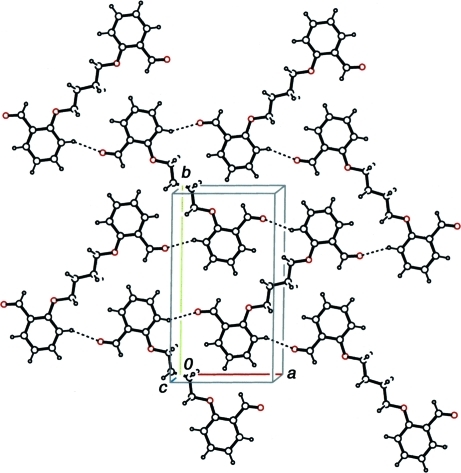
A packing diagram of the title compound, showing a molecular sheet formed by C—H···O hydrogen bonds (dashed lines).

## supplementary crystallographic information

### Comment

In recent years, much attention has been paid to the synthesis and coordination chemistry of salicylaldehyde, its Schiff base derivatives and transition metal complexes (Hu *et al.*, 2005; Aravindan *et al.*, 2003; Ilhan *et al*., 2009, 2010; Yilmaz *et al.*, 2009). The two-arm aldehydes can be condensed with primary diamines to form macrocyclic Schiff base ligands (Ilhan *et al.*, 2009, 2010; Yilmaz *et al.*, 2009).

In the crystal structure, the title molecule, 2,2'-[butane-1,4-diylbis(oxy)]dibenzaldehyde (Fig. 1), lies on a crystallographic inversion center, thus indicating that one half the molecule comprises the asymmetric unit. The molecules are linked through intermolecular C3—H3···O1^ii^ contacts (Table 1), resulting in a molecular sheet parallel to the (102) plane (Fig. 2).

### Experimental

The title compound was isolated from the reaction between salicylaldehyde and butane-1,4-diamine in the presence of K_2_CO_3_ at 85 °C for about 48 h according to the literature (Hu *et al.*, 2005). A small amount of the precipitate was dissolved in a mixture of methanol-chloroform (1:1 *v*/*v*) to make a clear solution and kept at room temperature for 3 days to give single crystals suitable for X-ray diffraction.

### Refinement

All H atoms were positioned geometrically (C—H = 0.95 or 0.99 Å) and were refined as riding, with *U*_iso_(H) = 1.2*U*_eq_(C).

### Crystal data

**Table:**

C_18_H_18_O_4_	*F*(000) = 316.00
*M**_r_* = 298.34	*D*_x_ = 1.306 Mg m^−^^3^
Monoclinic, *P*2_1_/*c*	Mo *K*α radiation, λ = 0.71075 Å
Hall symbol: -P 2ybc	Cell parameters from 7072 reflections
*a* = 8.0624 (7) Å	θ = 3.0–30.1°
*b* = 14.5896 (7) Å	µ = 0.09 mm^−^^1^
*c* = 6.8003 (4) Å	*T* = 190 K
β = 108.549 (4)°	Block, pale-yellow
*V* = 758.35 (8) Å^3^	0.30 × 0.24 × 0.15 mm
*Z* = 2	

### Data collection

**Table:**

Rigaku R-AXIS RAPID II diffractometer	1243 reflections with *I* > 2σ(*I*)
Detector resolution: 10.00 pixels mm^-1^	*R*_int_ = 0.041
ω scans	θ_max_ = 30.0°
Absorption correction: numerical (*NUMABS*; Higashi, 1999)	*h* = −11→11
*T*_min_ = 0.980, *T*_max_ = 0.986	*k* = −20→20
12149 measured reflections	*l* = −9→9
2210 independent reflections	

### Refinement

**Table:**

Refinement on *F*^2^	Primary atom site location: structure-invariant direct methods
Least-squares matrix: full	Secondary atom site location: difference Fourier map
*R*[*F*^2^ > 2σ(*F*^2^)] = 0.044	Hydrogen site location: inferred from neighbouring sites
*wR*(*F*^2^) = 0.132	H-atom parameters constrained
*S* = 1.13	*w* = 1/[σ^2^(*F*_o_^2^) + (0.0563*P*)^2^ + 0.0806*P*] where *P* = (*F*_o_^2^ + 2*F*_c_^2^)/3
2210 reflections	(Δ/σ)_max_ = 0.0001
100 parameters	Δρ_max_ = 0.29 e Å^−^^3^
0 restraints	Δρ_min_ = −0.24 e Å^−^^3^

### Special details

**Table:**

Geometry. All e.s.d.'s (except the e.s.d. in the dihedral angle between two l.s. planes) are estimated using the full covariance matrix. The cell e.s.d.'s are taken into account individually in the estimation of e.s.d.'s in distances, angles and torsion angles; correlations between e.s.d.'s in cell parameters are only used when they are defined by crystal symmetry. An approximate (isotropic) treatment of cell e.s.d.'s is used for estimating e.s.d.'s involving l.s. planes.
Refinement. Refinement of *F*^2^ against ALL reflections. The weighted *R*-factor *wR* and goodness of fit *S* are based on *F*^2^, conventional *R*-factors *R* are based on *F*, with *F* set to zero for negative *F*^2^. The threshold expression of *F*^2^ > σ(*F*^2^) is used only for calculating *R*-factors(gt) *etc*. and is not relevant to the choice of reflections for refinement. *R*-factors based on *F*^2^ are statistically about twice as large as those based on *F*, and *R*- factors based on ALL data will be even larger.

### Fractional atomic coordinates and isotropic or equivalent isotropic displacement parameters (Å^2^)

**Table:**

	*x*	*y*	*z*	*U*_iso_*/*U*_eq_	
O1	0.18122 (15)	0.33844 (8)	0.1640 (2)	0.0554 (4)	
O2	0.69626 (12)	0.36790 (7)	0.37956 (16)	0.0343 (3)	
C1	0.45915 (17)	0.26677 (9)	0.2840 (2)	0.0307 (3)	
C2	0.64045 (18)	0.27981 (9)	0.3598 (2)	0.0297 (3)	
C3	0.75273 (18)	0.20492 (10)	0.4082 (2)	0.0327 (3)	
H3	0.8758	0.2135	0.4596	0.039*	
C4	0.6825 (2)	0.11807 (10)	0.3803 (2)	0.0380 (4)	
H4	0.7586	0.0666	0.4134	0.046*	
C5	0.5042 (2)	0.10385 (11)	0.3054 (3)	0.0411 (4)	
H5	0.4582	0.0434	0.2868	0.049*	
C6	0.3940 (2)	0.17801 (10)	0.2582 (2)	0.0380 (4)	
H6	0.2712	0.1684	0.2071	0.046*	
C7	0.3391 (2)	0.34439 (11)	0.2358 (3)	0.0396 (4)	
H7	0.3882	0.4041	0.2625	0.047*	
C8	0.88087 (17)	0.38476 (10)	0.4570 (2)	0.0333 (3)	
H8A	0.9405	0.3547	0.3674	0.040*	
H8B	0.9307	0.3604	0.5996	0.040*	
C9	0.90444 (18)	0.48669 (10)	0.4558 (2)	0.0350 (4)	
H9A	0.8379	0.5159	0.5387	0.042*	
H9B	0.8568	0.5096	0.3117	0.042*	

### Atomic displacement parameters (Å^2^)

**Table:**

	*U*^11^	*U*^22^	*U*^33^	*U*^12^	*U*^13^	*U*^23^
O1	0.0243 (6)	0.0575 (8)	0.0782 (9)	0.0011 (5)	0.0074 (6)	−0.0063 (6)
O2	0.0210 (5)	0.0285 (5)	0.0497 (6)	−0.0040 (4)	0.0060 (4)	−0.0021 (4)
C1	0.0234 (7)	0.0324 (8)	0.0352 (7)	−0.0038 (5)	0.0077 (6)	−0.0030 (6)
C2	0.0268 (7)	0.0287 (7)	0.0333 (7)	−0.0051 (5)	0.0092 (6)	−0.0032 (6)
C3	0.0244 (7)	0.0320 (8)	0.0391 (8)	−0.0013 (6)	0.0065 (6)	−0.0020 (6)
C4	0.0358 (8)	0.0316 (8)	0.0447 (9)	0.0011 (6)	0.0102 (7)	0.0001 (7)
C5	0.0363 (8)	0.0317 (8)	0.0521 (9)	−0.0074 (6)	0.0095 (7)	−0.0039 (7)
C6	0.0286 (8)	0.0388 (9)	0.0441 (9)	−0.0085 (6)	0.0081 (7)	−0.0055 (7)
C7	0.0265 (8)	0.0372 (9)	0.0529 (10)	−0.0018 (6)	0.0095 (7)	−0.0027 (7)
C8	0.0206 (7)	0.0313 (7)	0.0453 (8)	−0.0026 (5)	0.0068 (6)	−0.0035 (6)
C9	0.0239 (7)	0.0294 (7)	0.0489 (9)	−0.0023 (6)	0.0075 (6)	−0.0035 (6)

### Geometric parameters (Å, °)

**Table:**

O1—C7	1.2128 (18)	C5—C6	1.372 (2)
O2—C2	1.3543 (16)	C5—H5	0.9500
O2—C8	1.4333 (16)	C6—H6	0.9500
C1—C6	1.3874 (19)	C7—H7	0.9500
C1—C2	1.3997 (19)	C8—C9	1.500 (2)
C1—C7	1.458 (2)	C8—H8A	0.9900
C2—C3	1.390 (2)	C8—H8B	0.9900
C3—C4	1.376 (2)	C9—C9^i^	1.516 (3)
C3—H3	0.9500	C9—H9A	0.9900
C4—C5	1.379 (2)	C9—H9B	0.9900
C4—H4	0.9500		
			
C2—O2—C8	118.21 (11)	C5—C6—H6	119.5
C6—C1—C2	118.83 (13)	C1—C6—H6	119.5
C6—C1—C7	119.95 (13)	O1—C7—C1	124.87 (14)
C2—C1—C7	121.21 (12)	O1—C7—H7	117.6
O2—C2—C3	123.48 (13)	C1—C7—H7	117.6
O2—C2—C1	116.14 (12)	O2—C8—C9	106.66 (11)
C3—C2—C1	120.37 (12)	O2—C8—H8A	110.4
C4—C3—C2	118.85 (13)	C9—C8—H8A	110.4
C4—C3—H3	120.6	O2—C8—H8B	110.4
C2—C3—H3	120.6	C9—C8—H8B	110.4
C3—C4—C5	121.62 (14)	H8A—C8—H8B	108.6
C3—C4—H4	119.2	C8—C9—C9^i^	111.50 (15)
C5—C4—H4	119.2	C8—C9—H9A	109.3
C6—C5—C4	119.28 (14)	C9^i^—C9—H9A	109.3
C6—C5—H5	120.4	C8—C9—H9B	109.3
C4—C5—H5	120.4	C9^i^—C9—H9B	109.3
C5—C6—C1	121.05 (14)	H9A—C9—H9B	108.0
			
C8—O2—C2—C3	−0.8 (2)	C3—C4—C5—C6	−0.2 (2)
C8—O2—C2—C1	179.88 (12)	C4—C5—C6—C1	0.2 (2)
C6—C1—C2—O2	179.34 (12)	C2—C1—C6—C5	−0.1 (2)
C7—C1—C2—O2	−1.8 (2)	C7—C1—C6—C5	−179.02 (15)
C6—C1—C2—C3	0.0 (2)	C6—C1—C7—O1	−3.4 (3)
C7—C1—C2—C3	178.91 (14)	C2—C1—C7—O1	177.73 (16)
O2—C2—C3—C4	−179.30 (13)	C2—O2—C8—C9	178.18 (12)
C1—C2—C3—C4	0.0 (2)	O2—C8—C9—C9^i^	177.46 (15)
C2—C3—C4—C5	0.1 (2)		

Symmetry codes: (i) −*x*+2, −*y*+1, −*z*+1.

### Hydrogen-bond geometry (Å, °)

**Table:**

*D*—H···*A*	*D*—H	H···*A*	*D*···*A*	*D*—H···*A*
C3—H3···O1^ii^	0.95	2.53	3.397 (2)	152

Symmetry codes: (ii) *x*+1, −*y*+1/2, *z*+1/2.
